# Implicit Block ACK Scheme for IEEE 802.11 WLANs

**DOI:** 10.3390/s16020167

**Published:** 2016-01-28

**Authors:** Pranesh Sthapit, Jae-Young Pyun

**Affiliations:** Department of Information and Communication Engineering, Chosun University, Gwangju, 501-759, Korea; pranesh@chosun.kr

**Keywords:** block ACK, IEEE 802.11e, overheads, MAC

## Abstract

The throughput of IEEE 802.11 standard is significantly bounded by the associated Medium Access Control (MAC) overhead. Because of the overhead, an upper limit exists for throughput, which is bounded, including situations where data rates are extremely high. Therefore, an overhead reduction is necessary to achieve higher throughput. The IEEE 802.11e amendment introduced the block ACK mechanism, to reduce the number of control messages in MAC. Although the block ACK scheme greatly reduces overhead, further improvements are possible. In this letter, we propose an implicit block ACK method that further reduces the overhead associated with IEEE 802.11e’s block ACK scheme. The mathematical analysis results are presented for both the original protocol and the proposed scheme. A performance improvement of greater than 10% was achieved with the proposed implementation.

## 1. Introduction

The explosion of various wireless networking technologies and smart devices are demanding the exchange of rich multimedia contents and sensed data [1,2,3,4,5]. As everyday devices are enabled with communication capabilities, we are entering into a new age of Internet of Things (IoT). IoT is expected to take us to a new era where everything is connected with the Internet. Therefore, the number of services and applications over IEEE 802.11 wireless LANs (WLANs) is rapidly increasing. As a device is expected to handle a large volume of data, power efficient policy is necessary for the long life of a network [6]. To support high data rates, IEEE 802.11 standard provides more than one data rates by employing multiple sets of modulation and channel coding schemes [7]. However, the transmission durations of control frames and inter-frame spaces are always of fixed value. In [8], the authors showed that there is an upper bound for throughput, and performance enhancements cannot be obtained by simply increasing the data rate, even to infinitely high levels, without reducing the overhead.

A new block ACK (BA) mechanism was presented in the IEEE 802.11e, to reduce MAC overhead [9]. A transmitter is assigned a fixed time duration called transmission opportunity (TXOP), during which a block of data frames is transmitted. Subsequently, the receiver aggregates the multiple ACK frames and sends a single BA that simultaneously acknowledges the status of all transmitted data frames by using a bitmap.

In IEEE 802.11n, the throughput enhancement is achieved by aggregating multiple packets before transmission [10]. Aggregation has several advantages. Aggregation reduces the channel waiting time during the backoff process for transmitting consecutive frames. In addition, the time used up in preamble and frame headers transmission are also saved. Even though, aggregation of multiple frames improves the network throughput under error free channel, a large aggregated frame means stations have to wait longer period of time for the channel access. Furthermore, under erroneous channel conditions, corruption of a large aggregated frame will waste substantial channel time leading to decreased MAC efficiency. Therefore, in the error-prone network environment, aggregation may result in lower MAC efficiency. Therefore, adaptive frame size is more suitable in a practical situation [11].

Thus, in this letter, we propose a scheme that can decrease the overhead of IEEE 802.11, without the use of aggregation techniques. We analyze the IEEE 802.11e BA scheme and propose an implicit BA request scheme. In addition, we discuss the proposed implicit BA scheme in detail and demonstrate how to improve IEEE 802.11e’s overall performance.

## 2. IEEE 802.11e

The MAC Service Data Unit (MSDU) is passed to IEEE 802.11e from upper layers. MSDU can be broken down into one or more MAC Protocol Data Units (MPDUs). In IEEE 802.11e, once a station successfully access the channel, the station is assigned a TXOP. During the TXOP, a station can transmit multiple MPDUs. TXOP is granted with both a start time and a maximum usable duration bounded by a threshold, referred to as the TXOP limit.

**Figure 1:**
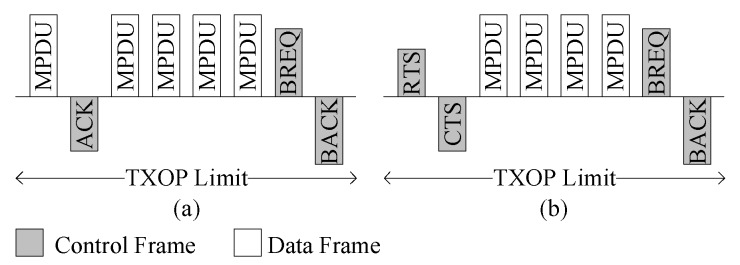
IEEE 802.11e block ACK in (**a**) basic access mode and (**b**) RTS/CTS access mode.

IEEE 802.11e introduced the BA scheme as shown in Figure 1 to reduce the overhead caused by multiple ACK transmissions. Basically, the BA allows multiple MPDUs transmission in a burst with a gap of short inter-frame space (SIFS) duration and acknowledged by BA frame (BACK). Upon receiving the BA request (BREQ) frame, the BACK frame is transmitted. BACK contains status regarding the success/failure reception of individual MPDU in the form of a bitmap. Transmission of both BREQ and BACK frames are done at data transmission rate. The transmitter and receiver must establish agreements before using the BA scheme. BA consists of setup and teardown phases. In the setup phase, capability information such as buffer size and BA policy are negotiated with the receiver. Similarly, the BA agreement is torn down with a so-called DELBA frame. Once BA setup is complete, multiple MPDUs can be transmitted in a block, with the expectation of a single BACK frame. After receiving the BACK, the sender retransmits the error MPDUs, either in the next TXOP or individually. The maximum limit of MPDUs that can be transmitted in the TXOP is defined in the setup phase. However, the number of MPDUs in a block is not dependent on the TXOP limit. The sender can divide the MPDUs that belongs to the same MSDU into multiple TXOPs. The BREQ can be sent in the same TXOP, or in the next TXOP. If there is no response until the expiration of the BREQ timer, the BREQ is retransmitted.

The head of burst (HOB) frame must be immediately acknowledged for the immediate detection of the collision. The RTS/CTS frame can be exchanged before starting communication for protecting the data burst. However, for the basic access modes, the first data frame is immediately acknowledged, as shown in Figure 1.

**Figure 2:**
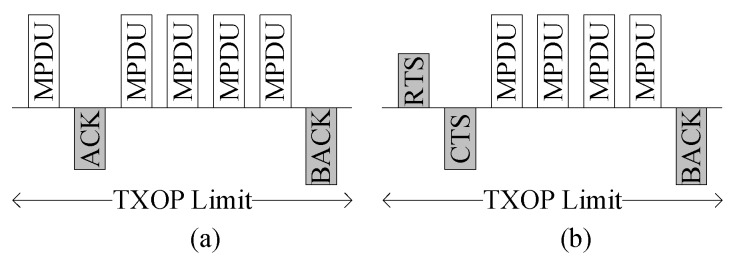
Proposed block ACK (BA) in (**a**) basic access mode and (**b**) RTS/CTS access mode.

## 3. Proposed Implicit Block Ack

In order to enhance the efficiency of BA in IEEE 802.11e, we propose an implicit BA request mechanism that decreases the overhead associated with BA.

### 3.1. Implicit Block ACK Request

The proposed implicit BA request scheme exploits the fact that the sender and receiver are aware of the presence of block transmissions during the TXOP period. Thus, we can simplify the block transmission by removing the BREQ frame, which consists of redundant information such as the MAC address and sequence number. In the proposed scheme, we do not use the explicit BREQ frame; the last MPDU serves as the implicit BA request. The removal of BREQ frames and their associated SIFS periods contributes to the BA efficiency enhancement. Figure 2 shows the proposed BA scheme implemented in both access modes. As seen in the figures, there are no explicit BREQ frames used. The last MPDU serves as the implicit BA request frame.

**Figure 3:**
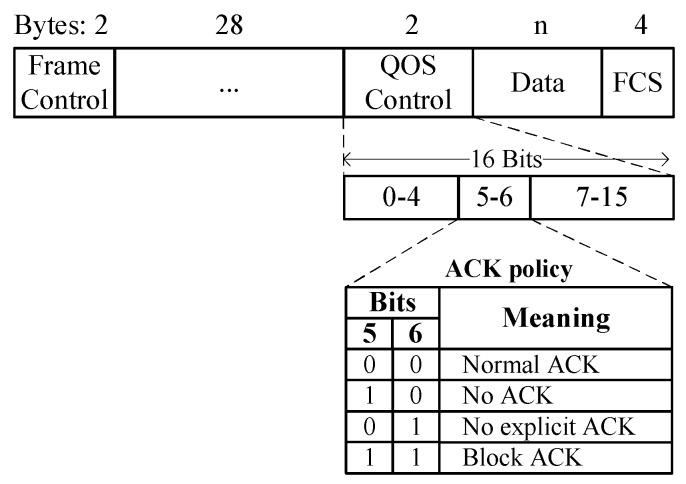
Frame format in IEEE 802.11.

The ACK policy subfield is included in the QoS control field of the IEEE 802.11 frame to indicate normal ACK policy or BA policy, as shown in Figure 3. In the legacy BA scheme, the ACK policy subfield is set to either normal ACK or block ACK during a transmission. In the proposed implicit BA request, the combination of both ACK policies is proposed. Once the stations agree on the BA mechanism, multiple MPDUs can be transmitted from the sender to the recipient for the TXOP period. All MPDUs, except the final one, are transmitted according to the block ACK policy. However, the final MPDU is transmitted using normal ACK policy. Thus, if the ACK policy field of MPDUs within a TXOP is transmitted with normal ACK policy, it implies a BA request. In other words, while using the BA mechanism, if the ACK policy of an MPDU is a normal ACK, the recipient will recognize it as the BA request and immediately send back the block ACK.

However, we do not propose the complete removal of the BREQ command. To manage BA timeouts, the BA request can still be used.

### 3.2. Re-Transmission of Damaged Frames

The BACK frame contains the status of all MPDUs received in the current TXOP in the form of a bitmap. Based on the bitmap, the sender retransmits the damaged or lost frames. However, the sender may occasionally experience a BA timeout. A BA timeout can occur in two situations: either the last MPDU is not received by the recipient, or the BACK frame is not received by the originator. The efficient way of handling the former case is to retransmit the last MPDU and the efficient solution for the latter case is to send BA request. Because the sender cannot recognize which frame in the two cases was lost during its transmission, we retransmit the last MPDU when a BA timeout occurs for the first time. However, if BA timeout occurs again because of transmission failure, then BREQ is sent.

## 4. Analytical Framework

We consider a network composed of *n* stations, and operate in saturated conditions. All stations are assumed to generate traffic with the same priority and have the same probability of winning channel. We assumed that source and destination are always fixed, thus, the BA setup phase overhead is omitted. Finally, we assume that stations experience uniform channel conditions, *i.e.*, they experience the same error rate. The discrete-time Markov chain of our model is same with the Markov chain employed in [12,13]. Furthermore, HOB refers to the data frame in the case of basic access or the RTS frame in the case of RTS/CTS access. Similarly, HACK is the HOB acknowledgment which is an ACK for the basic access and CTS for RTS/CTS access. Likewise, end of block (EOB) is the BREQ in the case of the original method, whereas EOB is the data frame in the case of the proposed method. When multiple stations try to access at the same slot, they contend via the random backoff procedure. Therefore, the probability *τ* that a station transmits in a randomly chosen slot time is expressed as derived in [13],

(1) $$\tau =\frac{2\left(1-2p\right)\left(1-p^{m+1}\right)}{CW_{min}\left(1-2p^{m^{\prime }+1}\right)\left(1-p\right)+\left(1-2p\right)\left[CW_{min}2^{m^{\prime }}p^{m^{\prime }+1}\left(1-p^{m-m^{\prime }}\right)+1-p^{m+1}\right]}$$

where $CW_{min}$ is the minimum backoff window size, *m’* is the maximum backoff stage, and *m* is the maximum retransmission limit. Each packet is transmitted with a header of *h* bytes. A packet is corrupted if at least one bit is received in error. Therefore, the probability of successful transmissions (PST) of all bits [14] is given by

(2) $$PST={\left(1-BER\right)}^{(k+h)\times 8}$$

A station is unable to access the channel because of collision or due to loss of HOB or HACK frame [15]. However, if HOB and HACK frames are successfully exchanged, then data transmission burst starts. The probability *p* that a packet collides or unsuccessful due to error is derived as

(3) $$p=1-{\left(1-\tau \right)}^{n-1}PST_{HOB}PST_{HACK}$$

where *PST${}_{HOB}$* and *PST${}_{HACK}$* are the frame success rates of HOB and HACK frames, respectively. Among *n* stations, the probability $P_{tr}$ that there is at least one transmission in a time slot is given by

(4) $$P_{tr}=1-{\left(1-\tau \right)}^{n}$$

Channel access is successful if both HOB and HACK frames are exchanged successfully. The probability that a channel access attempt is successful ($P_{s}$) and the probability that all control frames are transmitted successfully ($P_{succ}$ ) are expressed as

(5) $$P_{s}=\frac{n\tau {\left(1-\tau \right)}^{n-1}}{P_{tr}}PST_{HOB}PST_{HACK}$$

(6) $$P_{succ}=\frac{n\tau {\left(1-\tau \right)}^{n-1}}{P_{tr}}PST_{HOB}PST_{HACK}PST_{EOB}PST_{BACK}$$

where, *PST${}_{EOB}$* and *PST${}_{BACK}$*are the frame success rates of EOB and BACK frames, respectively. The probability $P_{e}$ that a HOB or HACK are received in error is given by

(7) $$P_{e}=\frac{n\tau {\left(1-\tau \right)}^{n-1}}{P_{tr}}\left\{\left(1-PST_{HOB}\right)+PST_{HOB}\left(1-PST_{HACK}\right)\right\}$$

Finally, the system throughput *S* can be easily expressed as

(8) $$S=\frac{P_{s}E\left[P_{b}\right]}{E[slot]}$$

where *E[P${}_{b}$]* represents payload bits and *E[slot]* represents the average duration of transmission. It is to be noted that *E[slot]* consists of two parts: the idle time because of channel backoff, and the channel usage time due to successful burst transmissions, channel errors, or collisions, *i.e.*,

(9) $$E\left[slot\right]=\sigma E\left[bo\right]+P_{succ}E\left[T_{s}\right]+\left(1-P_{s}-P_{e}\right)E\left[T_{c}\right]+P_{e}E\left[T_{e}\right]+P_{s}E\left[T_{f}\right]$$

where *σ* represents the slot time, *E[bo]* represents the average number of backoff slots used, *E[T${}_{s}$]* is the average time for transmission, *E[T${}_{c}$]* is the average time wasted in collision, *E[T${}_{e}$]* is the average error time of HOB frames and *E[T${}_{f}$]* is the average error time of EOB frames, respectively.

Let *r’* and *r* be the employed control frame and data rate in Mbps, respectively. Let *RTS*, *CTS*, *ACK*, *BREQ*, *BACK*, and *DATA* represent channel occupancy times of corresponding frames. Similarly, *P* is the payload. Common physical header is included in every frame transmitted. Therefore, *H${}_{PHY}$* should be added to every frame. Table 1 shows the time durations of all the relevant frames in *μ*s. In this analysis, we assume each TXOP always ends with a BACK transmission.

**Table 1:** Transmission time in *μ*s.

RTS	CTS	ACK	BREQ	BACK	DATA	H ${}_{\mathrm{PHY}}$
160/r’	112/r’	112/r’	192/r	1261/r	(224+P)/r	192

The station can transmit multiple MPDUs for the TXOP period. The channel occupancy time can be split into three parts: (i) overhead for the channel access *AO*, (ii) the data transmission burst, and (iii) overhead for the channel release *RO*. Table 2 shows the values of these components. The maximum number of MPDUs *d*, that can be transferred in a TOXP can be derived as

(10) $$d=\left\lfloor \frac{TXOP-AO-RO}{DATA+SIFS}\right\rfloor$$

**Table 2:** Decomposition of channel utilization times.

	AO	RO
**Basic access**	ACK+SIFS	BREQ+2SIFS+BACK
**Proposed basic access**	ACK+SIFS	SIFS+BACK
**RTS/CTS access**	RTS+2SIFS+CTS	BREQ+2SIFS+BACK
**Proposed RTS/CTS access**	RTS+2SIFS+CTS	SIFS+BACK

Once a station gains the channel, regardless of the error in a data frame, the station transmits all data frames. Therefore, the total transmission time is given by:

(11) $$E\left[T_{s}\right]=AO+d\times \left(DATA+SIFS\right)+RO-SIFS+DIFS$$

However, because of the transmission error, the number of successfully transmitted data frames can be less than *d*. The average number of data frames per data burst and overall payload are given by

(12) $$d_{e}=d\times PST_{DATA}$$

(13) $$E\left[P_{b}\right]=d_{e}\times P$$

Similarly, the average collision time experienced by a station can be expressed as:

(14) $$E[T_{c}]=HOB+EIFS$$

where *HOB* is the time utilized for the HOB transmission. The time wasted due to error is determined by the length of the corrupted frames. The error in HACK can only occur if the HOB frame is received successfully. Therefore, the average error time experienced by a station can be expressed as

(15) $$\begin{array}{c} E\left[T_{e}\right]=\left(1-PST_{HOB}\right)\left(HOB+HACK+EIFS\right)+PST_{HOB}\left(1-PST_{HACK}\right)\left(HOB+HACK+EIFS\right) \end{array}$$

Likewise, the average time wasted due to error in EOB or BACK frames is given by

(16) $$\begin{array}{c} E\left[T_{f}\right]=\left(1-PST_{EOB}\right)\left(E\left[T_{s}\right]-DIFS+EIFS\right)+PST_{EOB}\left(1-PST_{BACK}\right)\left(E\left[T_{s}\right]-DIFS+EIFS\right) \end{array}$$

where PST${}_{EOB}$ and PST${}_{BACK}$ are the frame success rates of EOB and BACK frames, respectively.

## 5. Numerical Results

The numerical results presented here is obtained from the derived analytical framework. IEEE 802.11b was utilized as the underlying PHY. Unless specified, the default values of data payload size is of 1 KB and TXOP is 20 ms.

**Figure 4:**
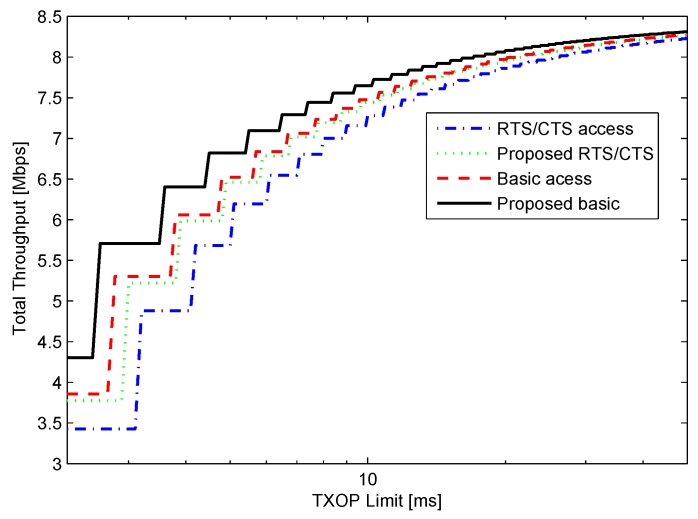
Throughput for *n* = 10, *r* = 1 Mbps, *r* = 11 Mbps, and bit error probability (BER) = 0.

Figure 4 depicts the network throughput for *n* = 10, *r’* = 1 Mbps, *r* = 11 Mbps, and BER = 0 with the various TXOP limits in both access modes. Note that the x-axis is in the logarithm scale. In the case of the error free channel, the minimum TXOP value is 2.2 ms; the time required for the transmission single MPDU (*i.e.*, *d* = 1). As the value of TXOP limit increases, the number of data unit that can be transmitted in a TXOP also increases. Figure 4 depicts a step increment whenever the number of MSDUs that can be transmitted in TXOP increases by one unit.

The figure depicts the significant performance improvement resulting from the implementation of the proposed method. The RTS/CTS and basic access modes with TXOP limit=2.2 ms give overall throughput equal to 3.42 Mbps and 3.85 Mbps, respectively. However, the proposed scheme is able to increase the throughput of RTS/CTS access and basic access modes to 3.77 Mbps and 4.3 Mbps, respectively. Because the number of contending stations is less, meaning less collision probability, the basic access mode exhibits better throughput performance than the RTS/CTS access mode.

**Figure 5:**
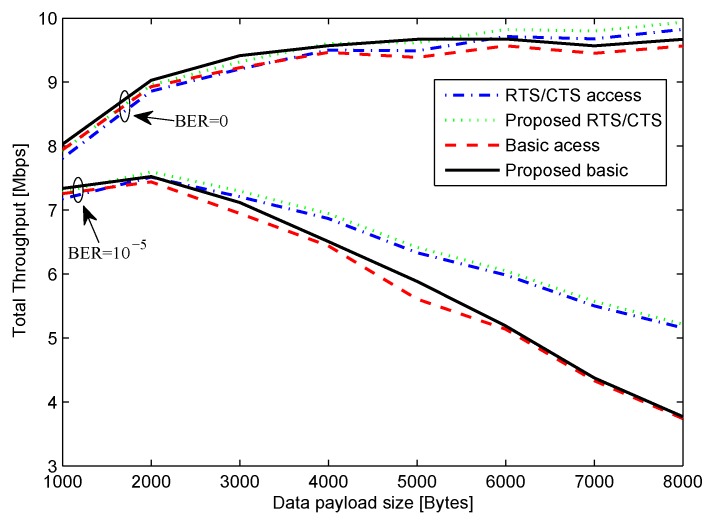
Throughput for *n* = 10, *r’* = 1 Mbps, *r* = 11 Mbps.

Figure 5 depicts the system throughput in the case of *n* = 10, *r’* = 1 Mbps, and *r* = 11 Mbps for various payload sizes. The graphs show the throughput for both ideal channel with BER=0 and for moderate error of BER = 10${}^{-5}$ when TXOP is 20 ms. In the case of BER = 0, as the payload size increases, the RTS/CTS method outperformed basics access method because of the high probability of collision in the case of basic access mode. Similar results were observed in the case of BER = 10${}^{-5}$. As the payload size increases, the probability of error also increases. The results verify that in the error-prone channel, larger payload size is not suitable [11]. Because HOB size of RTS/CTS access mode is much smaller as compared to basic access mode, RTS/CTS method exhibited better performance in the presence of channel error. The important observation is that the proposed method always gave better throughput than the original method. The reason for the performance improvement is because of decrement in the control packet overheads as compared to the original method.

**Figure 6:**
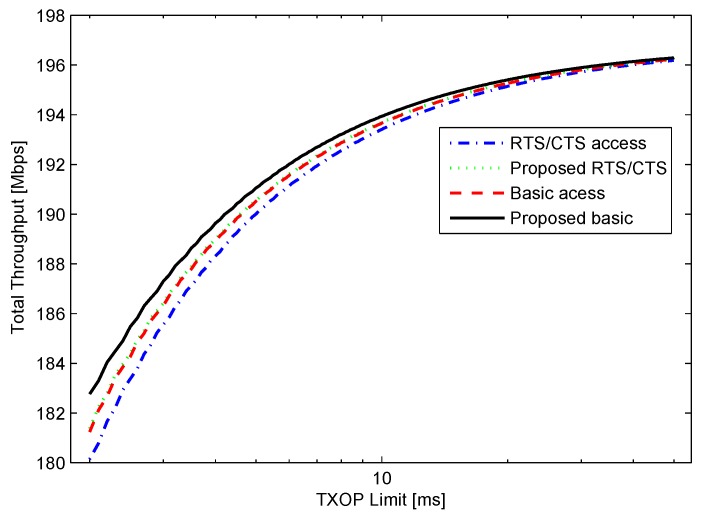
Throughput for *n* = 10, *r’* = 54 Mbps, *r* = 300 Mbps, and BER = 0.

To observe the performance for higher data rates under ideal channel condition, the basic rate, and data rate were set to 54 Mbps and 300 Mbps, respectively as in IEEE 802.11n. The obtained results are shown in Figure 6. As shown in the figure, the performance of the proposed BA scheme improves with the increase in data rate.

## 6. Conclusions

Network throughput is bounded by MAC overhead, even for infinitely high data rates. Therefore, reducing overhead is vital for achieving higher throughput. Even though, the legacy BA mechanism greatly reduces overhead, further improvements are still possible. In this letter, we proposed a method that reduces overhead associated with the BA scheme. The mathematical analysis results showed that the proposed scheme significantly improved throughput by more than 10%.
